# Zinc, vitamin A, and vitamin C status are associated with leptin concentrations and obesity in Mexican women: results from a cross-sectional study

**DOI:** 10.1186/1743-7075-9-59

**Published:** 2012-06-15

**Authors:** Olga Patricia García, Dolores Ronquillo, María del Carmen Caamaño, Mariela Camacho, Kurt Zane Long, Jorge L Rosado

**Affiliations:** 1 School of Natural Sciences, Universidad Autónoma de Querétaro, Av. De la Ciencia S/N, Juriquilla, Querétaro, 76230, Mexico; 2 Cindetec A.C., Jurica 122, Parque Industrial Querétaro, Querétaro, 76220, Mexico; 3 School of Population Health, University of Queensland, Herston Road, QLD 4006, Herston, Australia

**Keywords:** Micronutrient deficiencies, Obesity, Leptin

## Abstract

**Background:**

The prevalence of obesity among Mexican women is high and it could be related to micronutrient status. We evaluated in a cross-sectional study the associations of zinc and vitamins A, C and E concentrations with BMI, central adiposity, body fat and leptin concentration.

**Methods:**

Women aged 37 ± 7.5 years (n = 580) from 6 rural communities in Mexico were evaluated. Anthropometric measurements included weight, height, waist and hip circumference. A fasting blood sample was taken for the analysis of glucose, lipid profile, leptin, zinc, and vitamins A, C and E. Body composition was determined by DEXA (Hologic Mod Explorer).

**Results:**

The prevalence of overweight and obesity was 36% (BMI > 25 Kg/m^2^) and 44% (BMI > 30 Kg/m^2^), respectively. Prevalence of zinc and vitamins C and E deficiencies were similar in obese, overweight and normal weight women. No vitamin A deficiency was found. Vitamin C was negatively associated with BMI, waist-to-height ratio, and leptin concentrations (p < 0.05)*.* Vitamin A was positively associated with leptin (p < 0.05). When stratifying by BMI, % body fat and waist circumference, high leptin concentrations were associated with lower zinc and lower vitamin C concentrations in women with obesity (p < 0.05) and higher vitamin A concentrations in women without obesity (p < 0.01). Vitamin E status was not associated with any markers of obesity.

**Conclusion:**

Zinc and vitamins A and C are associated with obesity, adiposity and leptin concentration in women from rural Mexico, and may play an important role in fat deposition. The causality of these associations needs to be confirmed.

## Background

The association between micronutrient deficiencies and obesity is particularly important in populations where micronutrient deficiencies are widely widespread. About 30% of women in Mexico have zinc deficiency, 30% vitamin E and 50% are vitamin C deficient, and deficiencies are more widespread in rural areas [1]. Also, approximately 15% of women of reproductive age are anemic [2]. The combined prevalence of overweight and obesity in Mexican women is 69.3%, and it has increased more in rural (23%) than in urban (19%) areas in the past 7 years [3,4]. Therefore micronutrient deficiencies may coexist with obesity and obesity related diseases, in such a way that micronutrient deficiencies may be contributing to the increase of overweight and obesity observed in these populations.

Recent evidence suggests that deficiencies of some micronutrients are related to obesity and fat deposition [5]. Obese individuals have lower blood concentrations of some vitamins and minerals compared to non-obese individuals [6-8]. Micronutrient deficiencies may increase the risk of fat deposition and thus, of obesity and related diseases. The relationship between micronutrients and obesity might be affected by leptin, an adipokine associated with satiety. Vitamin C, for example, has been shown to inhibit leptin secretion and other glucose/lipid metabolic pathways, such as inhibiting glucose uptake and reducing glycerol release in animal models [9]. Retinoic acid reduces the expression and secretion of leptin in both, human and animal adipose tissue [10,11]. Vitamin E has been shown to decrease leptin expression in rats [12] and low vitamin E intake has been associated with the expression of genes associated with obesity, such as *SIRT1* variants [13]. It has been observed that zinc concentration is directly associated with serum leptin concentration [14], and supplementing with zinc decreases leptin in obese individuals [15].

We have found that supplementation of Mexican women from rural communities with low-fat milk added with micronutrients reduced body weight, Body Mass Index (BMI) and total body fat significantly more than a group supplemented with low-fat milk alone [16]. A similar study demonstrated that supplementing obese Chinese women with micronutrients plus calcium contributed to reduce body weight, body fat and improve lipid profile more than calcium supplementation alone [17]. Results from both studies suggest that improving micronutrient status might be important for the prevention of obesity and also to increase the effectiveness of obesity treatments in populations with high micronutrients deficiencies, high prevalence of obesity and high prevalence of obesity associated diseases.

The objective of the present study was to determine the relationship between nutritional status of zinc and vitamins A, C and E with BMI, visceral adiposity, total body fat and leptin concentrations as possible risk factors of obesity and chronic disease in women living in rural areas of Mexico.

## Methods

### Subjects and study design

A total of 580 adult women participated in this cross-sectional study. Women between 25 and 55 years of age from 6 rural communities in the state of Queretaro in Mexico (La Esperanza, La Peñuela, México Lindo, San Ildelfonso, San Vicente and Purísima de Cubos) were invited to participate in the study. The women received both oral and written information of the study. Women with uncontrolled hypertension, type II diabetes or that were pregnant or lactating were not included in the study. Those who voluntarily decided to participate signed an informed consent form. The study was approved by the Bioethics Committee of the Universidad Autonoma de Queretaro (UAQ).

The sample size was calculated considering an estimated prediction of BMI with micronutrients in a linear regression, with an estimated coefficient of 0.3 kg/cm^2^, a standard deviation of 2.15 μg/mL and the regression residuals’ standard deviation of 5.5 kg/cm^2^. Considering a two tailed significance level of 5% and a statistical power of 80%, the total sample size was 573 women*.*

Once enrolled, participants were asked to attend the health clinic at their communities and were interviewed to determine their socioeconomic status (SES) and dietary intake. During the same visit, a single fasting blood sample was collected from the participants for the determination of glucose concentration, lipid profile, leptin, zinc and vitamins A, C and E concentrations. These micronutrients were measured because of the high prevalence of deficiency previously reported in Mexico for women of the same age group [1] and based on the evidence linking these micronutrients to obesity [18].

### Anthropometry and body composition evaluations

On a separate day, participants were transported from their communities to the Metabolic Unit at UAQ for anthropometry and body composition evaluations. Weight, height, and waist and hip circumferences were measured in duplicate by trained personnel following standard procedures [19]. Women were weighed in light clothing using a digital scale (SECA Mod 843, Hamburg, Germany) with a precision of 0.1 g. Height was determined using a portable stadimeter (SECA Mod 206, Hamburg, Germany) with a 0.1 cm precision. For the present study, obesity was considered with a BMI ≥ 30 kg/m^2^, and overweight with a BMI 25 – 29.9 kg/m^2^[20]. Waist and hip circumferences were measured with a 0.1 cm precision using flexible fiber glass measuring bands (SECA Mod 200, Hamburg, Germany). High risk waist circumference was considered above 80 cm, and high risk waist-to-hip ratio above 0.85 [20]. The waist-to-height ratio was also calculated because some studies have considered that it could be a better indicator than BMI or waist circumference to identify individuals at risk of chronic disease [21]. High risk waist-to-height ratio was considered below 0.5 [22].

Whole body composition was measured by a certified technician using DEXA (Hologic Mod Explorer, 4500 C/W QDR, INC 35 Crosby Drive, Bedford, MA 01730, USA). Percent body fat, body fat content (Kg) and fat free mass (Kg) were determined directly from equipment measures; abdominal fat mass and abdominal fat percent were estimated following a procedure previously described by Hill et al. [23]. A normal fat percent was considered below 25%, moderate risk between 25 and 30%, and high risk >30% [2].

### Laboratory analysis

A fasting blood sample was collected by venipuncture from each subject on the first visit to the community health clinic. Women were instructed not to eat anything at least 12 hours before blood sample was collected early in the morning. Plasma was separated in blood samples by centrifugation at 1800–2000 rpm for 15 minutes and aliquots were stored at −70 °C for later analysis. Blood analysis included glucose, total cholesterol, low-density lipoprotein (LDL), high-density lipoprotein (HDL), triglycerides, leptin, vitamins A, C and E, and zinc. All laboratory analyses were performed in duplicate at the Human Nutrition Laboratory of UAQ.

Glucose, total cholesterol and triglycerides concentrations were determined in plasma with commercial kits (Elitech, Sees, France) using a clinical chemical analyzer (Bayer RA-50, Bayer Diagnostics, Dulin, Ireland). Both, HDL cholesterol and LDL cholesterol were measured by spectrophotometry (Genesis 20 ThermoSpectronic, Thermo Electron Corp, Madison, WI) with commercially available kits (Cholesterol HDL, Elitech, Sees, France; Cholesterol LDL, Spinreact, Sant Esteve de Bas, Spain). High glucose was considered with fasting glucose concentrations > 110 mg/dL, high total cholesterol with concentrations >200 mg/dL, high LDL with concentrations > 100 mg/dL, high triglycerides with concentrations >150 mg/dL, and low HDL with concentrations <50 mg/dL [24].

Plasma leptin concentration was quantified by a commercial kit using ELISA (Human Leptin Elisa Kit, Linco Research, St Charles, MO) using a microplate photometer (Multiskan Ascent, Thermo Electron Corporation, Vaanta, Finland).

Zinc concentrations were measured in duplicate by atomic absorption spectrophotometry (AAnalyst 7000, Perkin Elmer Instruments, Norwalk, CT). Zinc deficiency was defined with zinc plasma concentrations <70 mg/L [25]. HPLC was used to determine vitamins A, C and E. Vitamins A and E determination was based on the reported technique by Bieri et al. [26]. This technique consists of the simultaneous determination of both vitamins by reverse phase High-Pressure Liquid Chromatography (HPLC) (Waters Mod 2996, Waters Associates, Milford, MA), using the corresponding USP certified standards (retinol and alpha-tocopherol), with a C18 column (WATERS, New Braunfels, TX), and a mobile phase of 100% methanol (J.T.Baker). Both vitamins were measured at a wavelength of 300 nm. Vitamin A deficiency was considered with retinol concentrations <20 μg/dL [27]. Vitamin E deficiency was defined with vitamin E:lipids ratio < 1.6 μmol/mmol [27]. Vitamin C determination was made using a previously reported technique [28] by HPLC with a photodiode detector (Waters Mod 2996, Waters Associates, Milford, MA), using a C18 column (WATERS, New Braunfels, TX) and a mobile phase of NaH_2_PO_4_ 0.01 M and EDTA 0.2 mM; ascorbic acid was measured at a wavelength of 254 nm. Vitamin C deficiency was considered with blood concentrations of < 2 μg/mL and low vitamin C levels with <4 μg/mL [27].

### Dietary intake evaluation

Trained nutritionists assessed the usual diet in a subsample of the population studied (82 women) using three 24-hour recalls, one applied at a weekend day and two during other days of the week. Daily nutrient intake was calculated using food composition tables from the United States Department of Agriculture (USDA) [29] and from the National Institute of Medical Sciences and Nutrition “Salvador Zubirán” [30].

### Data analysis

The distributions of dependent variables in each analysis were explored to confirm their normal distribution. Leptin was transformed with square root to fit normal distribution for the analysis and coefficients were retransformed to its original units in the results. Women were classified according to their BMI into normal, overweight or obese, and main descriptive statistics were calculated for each BMI group and for the overall sample. Micronutrient concentrations were compared among BMI groups with one factor Analysis of Variance (ANOVA) and the Least Squared Difference (LSD) post-test was used to compare categories. To determine the association of micronutrient deficiencies with BMI groups the chi square test was performed. Linear regression was carried out to analyze the association of micronutrient concentrations with BMI, fat percent, abdominal fat and biochemical variables while adjusting for age, crowding and mother’s educational level. The association of micronutrient concentrations with leptin in the linear regression was then further explored stratifying by BMI groups, body fat percent and waist circumference tertiles. All data was analyzed using SPSS v 18 (SPSS, Inc., Chicago, IL)

## Results

General characteristics of the women that participated in the study are shown in Table 1. The prevalence of overweight and obesity was 36% and 44% respectively. Overall, 96% of the women had a body fat percent >30%, and 80% had a waist circumference >80 cm. Overweight and obese women had significantly higher LDL, triglycerides and leptin, and lower HDL concentrations than women with normal weight (p < 0.05). Approximately 22% of the women with obesity and overweight had high total cholesterol, 49% had high triglycerides, and more than 60% had low HDL concentrations.

**Table 1:** **Age, anthropometry, body composition, and biochemical variables of the women that participated in the study**^**1**^

**Variables**	**Normal weight (BMI ≤ 25Kg/m**^**2**^**)**		**Overweight (BMI > 25Kg/m**^**2**^**)**		**Obese** **(BMI > 30Kg/m**^**2**^**)**		**Overall**	
Age, years	**114**	**33.9 ± 6.8 ^a^**	**211**	**38.2 ± 7.5 ^b^**	**255**	**37.1 ± 7.6 ^b^**	**580**	**37.0 ± 7.6**
**Anthropometry**								
Body Mass Index, Kg/m^2^	114	22.9 ± 2.0 ^a^	211	27.5 ± 1.4 ^b^	255	34.5 ± 4.1 ^c^	580	29.7 ± 5.5
Waist circumference, cm	114	76.6 ± 5.2 ^a^	211	86.3 ± 4.9 ^b^	255	99.9 ± 9.0 ^c^	580	90.4 ± 11.5
Hip circumference, cm	114	93.4 ± 5.1 ^a^	211	99.5 ± 5.0 ^b^	255	112.8 ± 10.9 ^c^	580	104.2 ± 11.4
Waist to hip ratio	114	0.8 ± 0.1 ^a^	211	0.9 ± 0.1 ^b^	255	0.9 ± 0.1 ^c^	580	0.9 ± 0.1
Waist to height ratio	114	0.5 ± 0.4 ^a^	211	0.6 ± 0.3 ^b^	255	0.7 ± 0.6 ^c^	580	59.7 ± 8.0
**Body composition**								
Body fat, Kg	114	17.6 ± 3.0 ^a^	210	22.9 ± 3.0 ^b^	252	32.2 ± 6.3 ^c^	576	25.9 ± 7.5
Fat free mass, Kg	114	33.7 ± 2.9 ^a^	210	37.3 ± 3.3 ^b^	252	43.7 ± 4.8 ^c^	576	39.4 ± 5.6
Body fat, %	114	33.1 ± 3.6 ^a^	210	36.9 ± 3.1 ^b^	252	41.1 ± 3.6 ^c^	576	38.0 ± 4.6
Abdominal Fat, Kg	114	1.1 ± 0.3 ^a^	210	1.6 ± 0.3 ^b^	253	2.3 ± 0.6 ^c^	577	1.8 ± 0.7
Abdominal Fat, %	114	36.9 ± 5.1 ^a^	210	43.0 ± 3.8 ^b^	253	47.2 ± 4.1 ^c^	577	43.6 ± 5.7
**Laboratory Analyses**								
Glucose, mg/dL*	114	81.5 ± 10.0 ^a^	211	87.7 ± 29.0 ^b^	253	92.4 ± 30.2 ^b^	578	88.5 ± 27.2
Total cholesterol, mg/dL	114	153.2 ± 33.6 ^a^	211	168.6 ± 40.8 ^b^	252	168.4 ± 41.6 ^b^	577	165.5 ± 40.2
Low density cholesterol, mg/dL	113	77.2 ± 31.4 ^a^	204	87.9 ± 41.1 ^b^	246	92.2 ± 43.6 ^b^	563	87.6 ± 40.8
High density cholesterol, mg/dL	114	52.5 ± 12.2 ^a^	211	47.9 ± 13.5 ^b^	253	45.2 ± 11.0 ^c^	578	47.6 ± 12.5
Triglycerides, mg/dL	114	121.2 ± 73.6 ^a^	211	175.8 ± 124.9 ^b^	253	166.0 ± 87.6 ^b^	578	160.8 ± 102.5
Leptin, ng/mL *	113	16.1 ± 9.6 ^a^	210	25.8 ± 11.1 ^b^	254	44.7 ± 18.4 ^c^	577	32.2 ± 18.6

^1^ Values are mean ± standard deviation (SD). To convert total cholesterol, low density cholesterol, and high density cholesterol to SI units (mmol/L) multiply by 0.0259. To convert triglycerides to the international system (SI) units (mmol/L) multiply by 0.0113.

^a b c^ Different letters represent statistical significant differences in Posthoc Least Squared Difference (LSD) (p < 0.05).

* Analysis of variance (ANOVA) was performed with log-transformed data to achieve assumptions.

Dietary intake data of a subsample of the population is shown in Table 2. Overweight and obese women consumed significantly more calories and lipids than women with normal weight (p < 0.05). Intake of micronutrients was similar among groups.

**Table 2:** **Energy, macronutrient and zinc, vitamins A and C intake of the women that participated in the study (N = 82)**^1^

**Nutrient**	**Normal weight (BMI ≤ 25Kg/m**^**2**^**)**	**Overweight (BMI > 25Kg/m**^**2**^**)**	**Obesity** **(BMI > 30Kg/m**^**2**^**)**
Energy (Kcal)	1,480.3 (1,140.5, 1,820.1)^a^	1,767.7 (1,536.9, 1,998.6)^b^	2,014.2 (1,725.8, 2,302.5)^c^
Carbohydrates (g)	215.6 (23.5, 168.9)	258.8 (16.0, 227.0)	267.7 (19.9, 228.0)
Carbohydrates (%)	59.0 (53.8, 64.2)	58.4 (54.9, 62.0)	52.7 (48.3, 57.2)
Protein (g)	53.8 (7.3, 39.2)	59.0 (5.0, 49.1)	71.6 (6.2, 59.2)
Protein (%)	14.2 (12.4, 16.1)	12.9 (11.7, 14.2)	14.7 (13.2, 16.3)
Lipids (g)	46.4 (8.3, 29.9)^a^	58.9 (5.6, 47.7)^b^	76.6 (7.0, 62.6)^c^
Lipids (%)	26.8 (22.6, 31.0)	28.6 (25.8, 31.5)	32.5 (29.0, 36.1)
Zinc (mg) ^2^	5.6 (4.4, 6.8)	4.5 (3.7, 5.3)	5.7 (4.6, 6.7)
Vitamin A (μg) ^2^	739.1 (536.1, 942.0)	643.3 (508.1, 778.6)	663.5 (491.8, 835.2)
Vitamin C (mg) ^2^	70.0 (27.8, 112.1)	94.5 (66.4, 122.6)	53.9 (18.2, 89.5)
Vitamin E (mg) ^2^	16.7 (12.3, 21.2)	16.0 (13.1, 18.9)	17.7 (14.0, 21.5)

^1^ Values are means (95% Confidence Interval) from unadjusted analysis of variance (ANOVA).

^2^ Values are estimated mean (95%Confidence Interval) from analysis of covariance (ANCOVA) with energy as a covariable.

^a,b,c^ Different letters represent significant difference in Posthoc Least Squared Difference (LSD) test, p < 0.05.

### Vitamins and zinc status according to BMI groups

Zinc and vitamin C status and prevalence of deficiencies or low concentrations were similar in all BMI groups. Vitamin E concentrations were significantly lower in women with normal BMI than in women with obesity and overweight (p < 0.05) (Table 3). However, when adjusting for lipids (triglycerides and total cholesterol), vitamin E status was no longer different between groups. All women in the sample had adequate vitamin A status.

**Table 3:** **Zinc, vitamin A, vitamin C, and vitamin E concentrations in the women that participated in the study related to their Body Mass Index (BMI)**^1^

**Micronutrient**	**Normal weight (BMI ≤ 25Kg/m**^**2**^**)**		**Overweight (BMI > 25Kg/m**^**2**^**)**		**Obesity(BMI > 30Kg/m**^**2**^**)**		**Overall**		***p***^**2**^
Plasma zinc, μg/dL	109	1.2 ± 0.6	200	1.2 ± 0.7	243	1.3 ± 0.8	552	1.2 ± 0.7	0.624
Plasma zinc < 70 μg/dL (%)	19	17.4	33	16.5	45	18.5	97	17.6	0.856
Plasma ascorbic acid (Vitamin C), μg/mL	105	5.4 ± 2.2	194	5.4 ± 2.2	214	5.1 ± 2.2	513	5.3 ± 2.2	0.205
Plasma ascorbic acid < 4 μg/mL (%)	31	29.6	55	28.4	79	36.9	168	32.4	0.146
Plasma ascorbic acid < 2 μg/mL (%)	3	2.9	11	5.7	12	5.6	26	5	0.256
Plasma retinol (Vitamin A), μg/dL	113	46.2 ± 10.9	210	50.2 ± 10.8	250	48.2 ± 12.0	573	48.5 ± 11.4	0.100
Plasma retinol < 20 μg/dL (%)	0	0	0	0	0	0	0	0	*
Plasma retinol < 10 μg/dL (%)	0	0	0	0	1	0.4	1	0.2	*
Plasma a-tocopherol (Vitamin E), μg/mL	113	6.7 ± 1.9 ^a^	210	7.7 ± 2.1 ^b^	250	7.6 ± 2.2 ^b^	573	7.5 ± 2.2	<0.001
Vitamin E:lipids (μmol/mmol)	113	3.0 ±0.9	247	2.9 ± 0.9	570	2.9 ± 0.9	570	2.9 ± 0.9	0.547
Vitamin E:lipids < 1.6 μmol/mmol (%)	2	1.8	11	5.2	15	6.1	28	4.9	0.207

^1^ Values are mean ± standard deviation (SD) or %. To convert concentrations of vitamin C, vitamin A, vitamin E and zinc to international system (SI) units multiply by 56.78, 0.0349, 23.22 and 0.153, respectively (μmol/L).

^2^ Analysis of variance (ANOVA) or chi square significance.

^a b c^ Different letters represent statistical significant differences in Posthoc Least Squared Difference (LSD) (p < 0.05).

^*^ Chi square assumptions not met.

### Micronutrient status related to anthropometry, body composition and leptin

Vitamin C was negatively associated with leptin concentrations (p < 0.05) (Table 4). In addition, higher vitamin C concentrations were associated with lower BMI and waist to height ratio (p < 0.05). Vitamin A was positively associated with leptin concentrations (p < 0.05). Vitamin E concentration was positively associated with abdominal fat, BMI, waist circumference and waist-to-height ratio (p < 0.05). Vitamin E status adjusted for lipids was not associated with any of the anthropometrical or body composition variables.

**Table 4:** **Association of zinc and vitamins C, A and E concentrations with leptin concentration, body fat and anthropometry of the women that participated in the study**^**1**^

	**Vitamin C**	**Vitamin A**	**Vitamin E**	**Vitamin E:Lipids**	**Zinc**
	**(μg/mL)**	**(μg/dL)**	**(μg/mL)**	**(μmol/mmol)**	**(μg/dL)**
N	513	573	573	573	552
Leptin (ng/mL)^2^	−0.09 (−0.16, -0.01) *	0.02 (0.01, 0.04)*	−0.04 (−0.12, 0.05)	−0.10 (−0.28, 0.09)	−0.16 (−0.38, 0.09)
Body Fat (%)	−0.05 (−0.22, 0.13)	−0.01 (−0.05, 0.02)	0.15 (−0.03, 0.33)	−0.02 (−0.45, 0.40)	0.24 (−0.32, 0.80)
Abdominal Fat (Kg)	−0.02 (−0.04, 0.01)	0.00 (−0.01, 0.00)	0.03 (0.00, 0.05) *	0.00 (−0.06, 0.06)	0.05 (−0.03, 0.12)
Body Mass Index (Kg/m^2^)	−0.21 (−0.41, 0.00) *	−0.02 (−0.06, 0.02)	0.26 (0.05, 0.47) *	0.10 (−0.40, 0.61)	0.20 (−0.45, 0.85)
Waist circumference (cm)	−0.40 (−0.83, 0.03)	−0.01 (−0.09, 0.08)	0.51 (0.08, 0.95) *	−0.20 (−1.25, 0.84)	1.15 (−0.19, 2.50)
Waist-to-height ratio	−0.35 (−0.64, -0.05) *	−0.01 (−0.07, 0.04)	0.38 (0.08, 0.68) *	−0.09 (−0.80, 0.63)	0.92 (−0.01, 1.84)

^1^ Values are beta coefficients (95% Confidence Interval) from linear regression adjusted for years of age, crowding and years of mother's education. To convert concentrations of vitamin C, vitamin A, vitamin E and zinc to the international system (SI) units multiply by 56.78, 0.0349, 23.22 and 0.153, respectively (μmol/L).

^2^ Leptin was transformed with square root to fit normal distribution for analysis and coefficients were re-transformed to its original units. Leptin was also adjusted for fat percent.

* p < 0.05.

Anthropometry, body composition and leptin concentration data was also analyzed dividing the population according to their vitamin and zinc status, in deficient, non-deficient, and low concentrations. No differences were found between women with micronutrient deficiencies or low concentrations, and women with adequate micronutrient status, in any of the variables studied (data not shown).

### Association between micronutrient and leptin concentrations at different degrees of obesity

The analysis of the associations between micronutrients and leptin concentrations according to their BMI, and percent fat and waist circumference tertiles are shown in Table 5. When stratifying by BMI and body fat percent, zinc and vitamin C concentrations were negatively associated with leptin concentrations in women with obesity and with 36-40% body fat (p < 0.05). In addition, zinc was negatively associated with leptin in women with more than 94.3 cm of waist circumference (p < 0.05). Higher vitamin A concentrations were associated with higher leptin concentrations in women with normal BMI and with overweight, and women with <36% of body fat and <84 cm of waist circumference (p < 0.01).

**Table 5:** **Association of leptin concentration with vitamins A, C, E and zinc in the women that participated in the related to Body Mass Index (BMI), body fat and waist circumference terciles**^**1**^

	**Vitamin C**	**Vitamin A**	**Vitamin E**	**VitaminE:Lipids**	**Zinc**
	**(μg/mL)**	**(μg/dL)**	**(μg/mL)**	**(μmol/mmol)**	**(μg/dL)**
N	497	557	557	554	535
**Body Mass Index**					
≤ 25 Kg/m^2^	0.10 (−0.06, 0.26)	0.04 (0.01, 0.07)**	−0.03 (−0.19, 0.15)	0.04 (−0.29, 0.45)	0.60 (−0.07, 1.44)
> 25 Kg/m^2^	−0.07 (−0.19, 0.05)	0.03 (0.01, 0.06)**	−0.05 (−0.17, 0.08)	−0.10 (−0.36, 0.20)	0.01 (−0.34, 0.44)
> 30 Kg/m^2^	−0.14 (−0.26, -0.01)*	0.01 (−0.02, 0.03)	−0.09 (−0.21, 0.03)	−0.22 (−0.48, 0.08)	−0.41 (−0.66, -0.10)*
**Body fat terciles**					
≤ 36%	−0.02 (−0.14, 0.11)	0.05 (0.02, 0.07)**	0.04 (−0.09, 0.17)	−0.16 (−0.41, 0.14)	0.13 (−0.30, 0.68)
36 - 40%	−0.15 (−0.29, -0.01)*	0.02 (−0.01, 0.05)	−0.05 (−0.18, 0.09)	0.00 (−0.33, 0.40)	−0.40 (−0.67, -0.06)*
> 40%	−0.07 (−0.21, 0.07)	−0.01 (−0.04, 0.02)	−0.10 (−0.26, 0.06)	−0.14 (−0.45, 0.23)	0.15 (−0.34, 0.76)
**Waist circumference terciles**					
≤ 84.6 cm	0.04 (−0.08, 0.16)	0.04 (0.02, 0.07)**	0.05 (−0.09, 0.19)	0.14 (−0.15, 0.46)	0.03 (−0.36, 0.51)
84.6 – 94.3 cm	−0.06 (−0.20, 0.09)	0.03 (0.00, 0.05)	−0.03 (−0.16, 0.10)	−0.20 (−0.47, 0.13)	0.13 (−0.31, 0.69)
> 94.3 cm	−0.11 (−0.24, 0.03)	0.00 (−0.03, 0.03)	−0.11 (−0.24, 0.03)	−0.14 (−0.44, 0.23)	−0.42 (−0.69, -0.09)*

^1^ Leptin was transformed with square root to fit normal distribution for analysis and coefficients were re-transformed to its original units.

^2^ Values are beta coefficients (95% Confidence Interval) from linear regression adjusted for years of age, crowding, years of mother's education and fat percent, for regressions stratified by body fat percentiles which were adjusted just for age. To convert concentrations of vitamin C, vitamin A, vitamin E and zinc to International System (SI) units multiply by 56.78, 0.0349, 23.22 and 0.153, respectively (μmol/L).

* p < 0.05; ** p < 0.01.

## Discussion

In the present study, low vitamin C concentrations were associated with obesity and with higher leptin concentrations. In contrast, high vitamin A concentrations were associated with high leptin concentrations. When stratifying, high leptin concentrations were associated with lower zinc and vitamin C concentrations in women with obesity and with high body fat percent, and were also associated with higher vitamin A concentrations in women without obesity. Thus, zinc, and vitamins A, and C are associated with obesity, adiposity and leptin concentrations in women from a rural population in Mexico.

Zinc, vitamin A, vitamin C and vitamin E status of overweight and obese women were very similar to women with adequate BMI. These results agree to what has been previously reported, and results from these studies vary depending on the micronutrient studied. It has been observed, for example, that obese individuals have lower vitamin C concentrations, while vitamin E concentrations were normal compared with lean individuals [31]. Adult obese women were found to have significantly higher gamma-tocopherol concentrations compared with normal weight women, but alpha-tocopherol concentrations did not differ between groups [32]. Obese hypertensive patients in Poland had lower levels of hair zinc, but had similar serum zinc concentrations, compared with healthy adults [33]. Similarly, no differences were observed in zinc concentrations between obese and non-obese Turkish children [34]. Other studies have found differences in micronutrient status between obese and non-obese individuals. Low vitamins A and E concentrations, for example, have been found in morbidly obese Spanish patients [7], and low vitamin E and C concentrations in morbidly obese patients in Norway [6]. Thus, several factors might be involved that make the association between micronutrient status and obesity, inconsistent.

In the population studied, no differences were observed in the concentrations of vitamins A, C, E and zinc in the women according to their BMI, and this could be explained by their dietary habits. Overweight and obese women in this population had significantly higher intakes of energy and lipids compared with normal weight women. However, intake of micronutrients was similar in overweight, obese and normal weight women. This could be explained because obese individuals included several foods which are energy dense.

Low vitamin C concentrations were associated with higher BMI and higher waist-to-height ratio. Our results are similar to Johnston et al. [35] who found an inverse relationship between vitamin C and BMI and waist circumference in adults. The relationship observed between vitamin C and obesity in the women that participated in the study could have been caused by the effect that vitamin C has on leptin expression. Lower vitamin C concentrations were found to be associated with higher leptin concentrations in all women, and in obese women after stratifying by BMI. It has been observed that vitamin C dose-dependently inhibits leptin secretion in primary rat adipocytes [9]. In addition, vitamin C supplementation reduces the gene expression of apelin, an adipokine associated with insulin resistance, obesity and increased inflammation in animal models [36]. Higher concentrations of apelin are related to high concentrations of leptin and proinflammatory cytokines. Also, vitamin C has been shown to modify the response from rat adipocytes to high glucose levels, and to modulate the interaction between adipocytes and macrophages, protecting the adipocyte from a high glucose environment and from the inflammatory response associated with obesity [37,38].

Vitamin A is known to actively participate in the adipocyte metabolism. One of its metabolites, all-trans retinoic acid, has been known to stimulate lipolysis by activating the peroxisome proliferation-activated receptor delta and retinoic acid receptor [39,40]. Retinoic acid has been found *in vitro* to decrease preadipocyte survival time and to inhibit or promote adipose cell differentiation [41]. In addition, retinoic acid has been shown to inhibit the expression of leptin, resistin and uncoupling proteins (UCP) in mice and human cell culture tissues [10,11,41]. In our study, vitamin A concentrations were associated with high concentrations of leptin in the overall population, and the same was observed in women that had low BMI, low body fat percent and low waist circumference. These results suggest that vitamin A and its metabolites have different effects on leptin expression among individuals that differ in adipose tissue and total body fat content.

The association of zinc with obesity and body composition is not consistent. In obese Chilean children, for example, no association was found between zinc status and body composition [42]. In adults living in urban India, low concentrations of zinc were associated with higher abdominal fat [8]. In our study, zinc was not associated with obesity or leptin concentrations in the overall population. However, when stratifying by BMI and percent body fat, a negative association was found between zinc and leptin concentrations in women with obesity, with a body fat content of 36-40% and high waist circumference. This association could be explained by the effect of zinc-alpha2-glycoprotein (ZAG) on leptin concentrations. ZAG is an adipokine involved in the metabolism of lipids in the adipocyte that is down-regulated in obesity, probably due to the inflammation process associated with obesity. In obese individuals, low ZAG gene expression is associated with low serum adiponectin and high plasma leptin levels, and may play an important role in the development of obesity [43,44]. The influence zinc has on the adipocyte through the expression of leptin, by promoting free fatty acid release and glucose uptake, may also be controlled through the expression of a number of zinc-transporters in the adipocyte, that may be altered in obesity [45,46].

This is the first study to evaluate the relationship between zinc and vitamins A, C, and E, with leptin in women. Our findings suggest that the effects of vitamins and zinc concentrations in the adipocyte are complex and change depending on BMI, total body fat, and abdominal fat.

A major limitation of this study is that cross-sectional studies cannot establish causality. More studies are needed to understand the causes and consequences of micronutrient status on obesity and comorbidities. In addition, no vitamin A deficiency was found, and the prevalence of zinc, vitamin C and vitamin E deficiency was low. Thus, the relationship of vitamins A, C, E, and zinc with leptin could be different in populations with a high prevalence of these micronutrients deficiencies.

## Conclusions

In conclusion, in women living in rural Mexico, vitamin C and zinc concentrations were positively associated with measures of obesity and adiposity while vitamin A had the opposite effect. These micronutrients may be playing an important role in fat deposition and the pathogenesis of obesity. Future research should focus on studying causality and the effect of supplementation with multiple micronutrients on obesity in randomized clinical trials in populations with a high prevalence of micronutrient deficiencies.

## Abbreviations

ANOVA, Analysis of variance; BMI, Body mass index; DEXA, Dual-energy X-ray absorptiometry; ELISA, Enzyme-linked immunosorbent assay; EDTA, Ethylene-diamine-tetra-acetic acid; HDL, High-density lipoprotein; HPLC, High pressure liquid chromatography; LDL, Low-density lipoprotein; LSD, Least significance test; Rpm, Revolutions per minute; SES, Socioeconomic status; SPSS, Statistical package for social sciences; USDA, United States Department of Agriculture; USP, United States Pharmacopiea; UAQ, Universidad Autónoma de Querétaro; ZAG, zinc-alpha2-glycoprotein.

## Competing interests

The authors declare that they have no competing interests.

## Authors’ contribution

OPG contributed with the concept and study design, study implementation, and writing of the manuscript; DR coordinated all the field work and data collection; MCC participated in the data analysis and interpretation; MCB carried out all the biochemical analysis; KZL contributed to the study design, data analysis and interpretation. JLR contributed to concept and study design and provided important intellectual content to the manuscript. All authors read and approved the final manuscript.
